# Combining different diagnostic studies of lymphatic filariasis for risk mapping in Papua New Guinea: a predictive model from microfilaraemia and antigenaemia prevalence surveys

**DOI:** 10.1186/s41182-018-0123-8

**Published:** 2018-12-04

**Authors:** Alvaro Berg Soto, Zhijing Xu, Peter Wood, Nelly Sanuku, Leanne J. Robinson, Christopher L. King, Daniel Tisch, Melinda Susapu, Patricia M. Graves

**Affiliations:** 1 Information Resources, James Cook University, Townsville, QLD 4811 Australia; 2 Research School of Population Health, Australian National University, Canberra, ACT 2601 Australia; 3 College of Public Health, Medical and Veterinary Sciences, James Cook University, Cairns, QLD 4870 Australia; 4 Vector Borne Diseases Unit, PNG Institute of Medical Research, Goroka, Papua New Guinea; 5 Disease Elimination Program, Burnet Institute, Melbourne, VIC 3004 Australia; 6 School of Medicine and Veterans Affairs Administration, Case Western Reserve University, Cleveland, OH 44106 USA; 7 Department of Population and Quantitative Health Science, Case Western Reserve University, Cleveland, OH 44106 USA; 8 Malaria and Vector Borne Diseases, Public Health, Department of Health, Port Moresby, Papua New Guinea

**Keywords:** Lymphatic filariasis, Papua New Guinea, Prevalence, Predictive model, Diagnostic tests, Risk map

## Abstract

**Background:**

The Global Programme to Eliminate Lymphatic Filariasis has encouraged countries to follow a set of guidelines to help them assess the need for mass drug administration and evaluate its progress. Papua New Guinea (PNG) is one of the highest priority countries in the Western Pacific for lymphatic filariasis and the site of extensive research on lymphatic filariasis and surveys of its prevalence. However, different diagnostic tests have been used and thresholds for each test are unclear.

**Methods:**

We reviewed the prevalence of lymphatic filariasis reported in 295 surveys conducted in PNG between 1990 and 2014, of which 65 used more than one test. Results from different diagnostics were standardised using a set of criteria that included a model to predict antigen prevalence from microfilariae prevalence. We mapped the point location of each of these surveys and categorised their standardised prevalence estimates.

**Results:**

Several predictive models were produced and investigated, including the effect of any mass drug administration and number of rounds prior to the surveys. One model was chosen based on goodness of fit parameters and used to predict antigen prevalence for surveys that tested only for microfilariae. Standardised prevalence values show that 72% of all surveys reported a prevalence above 0.05. High prevalence was situated on the coastal north, south and island regions, while the central highland area of Papua New Guinea shows low levels of prevalence.

**Conclusions:**

Our study is the first to provide an explicit predictive relationship between the prevalence values based on empirical results from antigen and microfilaria tests, taking into account the occurrence of mass drug administration. This is a crucial step to combine studies to develop risk maps of lymphatic filariasis for programme planning and evaluation, as shown in the case of Papua New Guinea.

**Electronic supplementary material:**

The online version of this article (10.1186/s41182-018-0123-8) contains supplementary material, which is available to authorized users.

## Background

Lymphatic filariasis (LF) is a mosquito-transmitted disease caused by a parasitic nematode (predominantly *Wuchereria bancrofti*) that can seriously damage lymphatic vessels [1]. This frequently leads to cases of acute and chronic lymphoedema (extreme inflammation of lower limbs) and hydrocoele (swollen scrotum in men), potentially resulting in life-long chronic morbidity [2–4]. Unfortunately, these deformities are associated with the stigma of disfiguration and can lead to social isolation, economic hardships and mental distress [5].

Since the resolution to implement the Global Programme to Eliminate Lymphatic Filariasis (GPELF) by the World Health Assembly [6], the estimated global burden of LF has significantly decreased by 59% between 2000 and 2013 [7]. Despite this amazing achievement, about 68 million individuals in the world remain affected [7], with the corresponding DALYs (disability-adjusted life years lost) estimated at 2.02 million [8]. However, this estimation does not include disability from cases of mental illness resulting from stigmatising conditions, which was estimated at 5.09 million DALYs based on 2010 GBD data [5]. These numbers are of concern, suggesting that the GPELF must continue its strategy of annual single-dose mass drug administration (MDA) programs [1] to reduce the burden of lymphatic filariasis in the world.

Noteworthy are the efforts of the Pacific Program to Eliminate Lymphatic Filariasis (PacELF), a WHO initiative with island countries, territories and communities in the Western Pacific region, to collaboratively eliminate the disease from their populations [9]. This elimination process requires years of documentation, including initial mapping surveys, transmission assessment surveys (TAS) to evaluate MDA cessation and post-MDA surveillance for a period of at least 5 years [10]. Despite these complexities, the PacELF has achieved a measurable success towards their 2020 goals [9], with Vanuatu, Niue, Republic of Marshall Islands, Cook Islands and Tonga achieving official elimination of the disease [9]. Other countries, however, still face challenges [11]. Unfortunately, studies of filariasis in the region have been patchy and concentrated in only certain countries [12].

This patchy survey effort extends to Papua New Guinea (PNG), which continues to struggle with the disease. For instance, original mapping reported PNG as potentially containing one of the highest prevalence of lymphatic filariasis in the world [13], but there are in fact relatively large areas that are non-endemic to filariasis [14]. Although some studies show that filariasis prevalence has been reduced after MDA implementation in selected provinces (i.e. Western and Southern Highlands, West and East Sepik, Madang and New Ireland), these have been conducted in limited areas and usually for just a few years [15–19].

One of the most critical challenges in the Western Pacific region is to identify new strategies to scale up MDA in PNG [20, 21]. Localised MDA implementation for areas with high endemicity in PNG has been proposed as a starting strategy [14], which would benefit from reliable mapping based on more localised information on disease distribution. However, previous work summarising surveys conducted in the country from 1980 to 2011 exposed the many challenges of extracting useful information from unrelated studies [14]. For instance, previous survey efforts in PNG used different diagnostic techniques to report their prevalence [14], as new diagnostic techniques emerged over time [22]. Nevertheless, the large amount of information obtained in these surveys from PNG can help us understand the relationship between prevalence estimates obtained by different diagnostic methods.

Diagnostic tests used in PNG generally fall into two categories:(a) microfilaraemia (Mf) detection through blood slides and (b) antigen tests through either point-of-care immunochromatographic test cards (ICT) or laboratory analysis (Og4C3 ELISA test) [23]. The first type of diagnostics detects early-stage nematode worms circulating in the blood stream [23]. In the case of Papua New Guinea, these microfilariae follow a nocturnal circulating cycle, to match the feeding behaviour of the main vector in the region, *Anopheles* mosquitoes [24]. The two other diagnostic tests detect antigen from the adult worms instead [23]. Efforts to understand the relationship between these types of diagnostics have been inconclusive [25, 26] but remain crucial for combining varying types of diagnostic results to develop risk maps of LF in the region [26]. Thus, the association between Mf detection and antigen results remains an important research question.

Few studies have explored this association, generally concluding that these two variables lack a predictive relationship that could be used to combine results in studies [25]. While efforts using logistic regression were unable to produce a predictive model [25], further approaches have proposed that a relationship between these two diagnostic types exists, and it is based on the distribution of adult worms and their Mf output [26]. These studies suggest that the relationship between Mf and antigen diagnostics changes with the presence of MDA deployment [25, 26], as its implementation effectively reduces the number of Mf, but it takes time to kill off the adult worms which continue to produce antigen in the bloodstream. This is an important aspect to consider when developing models to describe this relationship from surveys conducted before and after MDA.

To develop a predictive model between LF diagnostics tests, we re-examined previously reviewed data summarised only at the district level [14] and consolidated all existing surveys conducted in PNG since 1990. We used this first explicit predictive model of its kind to aggregate empirical results from different diagnostic tests and refine our knowledge of LF distribution according to point estimates of survey locations. In this paper, we examined the attributes of our proposed model and its practical use to improve the accuracy of LF risk maps in PNG.

## Methods

### Survey selection

We reviewed a total of 312 survey results originally summarised by Graves et al. [14]. Each of these surveys conducted a cross-sectional blood survey for LF, surveying either one or more villages, schools or a main city and its catchment. These studies used different diagnostic methods, as described above, covering 80 different districts, from 1980 to 2011 [14]. In the current study, we restricted the surveys to those conducted from 1990 onwards, as explained below. In addition, we included an extra 14 more surveys that were conducted more recently in 2014. We also extracted information on the number of MDA rounds (i.e. DEC and albendazole) performed prior to each survey.

### Prevalence standardisation

We developed a set of criteria to standardise different diagnostic results from surveys into one chosen type. Following PacELF monitoring strategies [9], we chose to standardise for antigen prevalence values over Mf detection, as the former can be used independently of the diurnal/nocturnal cycles of microfilariae [23]. As antigen tests can determine the presence of adult worms and the potential for ongoing transmission even after MDA programs, this type of diagnostics is highly appropriate for the purpose of MDA implementation and culmination [23].

To standardise the results in favour of antigen prevalence values, we did the following:

1. As the first reported LF prevalence survey in PNG using an antigen test (i.e. Og4C3 ELISA) was in 1990, all surveys previous to this year were not included in our study.
2. For surveys reporting prevalence values from either an ICT or an Og4C3 ELISA test, these values were considered without further modifications.
3. For surveys reporting values from both Mf counts and one other antigen test, only values from the antigen diagnostics were considered for the final mapping, although these surveys were used to develop our predictive model (see point 6).
4. For surveys that reported values from both ICT and Og4C3 ELISA tests together, the average was calculated.
5. When a combination of all three diagnostics was used in a survey, only the average of the two antigen tests was considered.
6. For surveys that reported prevalence value based on microfilariae detection only, we predicted antigen values from these based on a predictive model developed specifically for this study. This process is described in the following section.

### Predicting antigen prevalence values from Mf estimates

We collected prevalence values from surveys that reported both Mf and antigen prevalence results (surveys in points 3 and 5 above). While the majority of these 65 surveys have been published in the peer-reviewed literature, some were extracted from PhD theses and others from unpublished reports by the WHO or by PNG Department of Health. Data from 51 of these surveys were previously summarised and reported in detail by Graves et al. [14]. The remaining 14 surveys were recently conducted by authors of this paper in the East and West Sepik provinces as part of ongoing clinical trials evaluating MDA annual dosages. While procedures followed in these selected surveys varied, they were all performed according to best practice recommended techniques with oversight from the relevant funding bodies, academic institutions and/or publishers.

Considering previous indications that the relationship between Mf and antigen diagnostics changes with the presence of MDA [26–28], we began by averaging ICT and Og4C3 values for surveys that reported more than one antigen test. We then conducted an ANOVA test between prevalence values from surveys conducted before and after any MDA, for both Mf and antigen results separately. This was performed to test for a significant difference with the presence/absence of MDA that would preclude the investigation of our predictive model.

Through regression analyses, we evaluated four different models of the potential relationship between tests that took into consideration a possible MDA interaction. These models were based on exponential or power functions grounded on observed patterns in the data. We investigated two types of MDA interaction for each model explored: (a) presence/absence (*δ* = 1 for post-MDA) and (b) number of MDA rounds. We also developed two sets of considerations in the analyses of these models based on two possible scenarios:

(A) *Differentiated Scenario models*: This approach assumed that a different type of function had to be applied as a response to the interaction. Thus, the dataset was categorised into two groups: pre- and post-MDA surveys. Individual functions were tested, and the optimum models were determined separately for the pre- and post-MDA data (see Table 1).

**Table 1:** Comparison of the Differentiated Scenario A models

Scenario A Model functions		Pre-MDA		MDA interaction	Post-MDA	
		RSE	AIC		RSE	AIC
1	*y* = *a*(1 − *e*^−*bx*^)	0.1187	**− 65.86**	*δ*	0.1789	− 6.69
				*N*	0.1991	− 3.27
2	*y* = *a* − *be*^−*cx*^	0.1192	− 64.49	*δ*	0.1603	− 10.20
				*N*	0.1885	− 5.03
3	*y* = *ae*^−*b*/*x*^	0.1218	− 63.30	*δ*	0.2036	− 2.56
				*N*	0.2198	− 0.11
4	*y* = *ax*^*b*^	0.1209	− 64.04	*δ*	0.1400	**− 14.53**
				*N*	0.2024	− 2.74

Differentiated Scenario A models evaluated, with their corresponding RSE and AIC values. The *y* variable represents the predicted antigen estimates, while *x* is the Mf prevalence as the independent variable. *δ* is the presence of MDA while *N* is the rounds of MDA (acting as indicators). Numbers in bold represent the lowest AIC values for pre- and post-MDA conditions, suggesting the best-fit models
(B) *Combined Scenario models*: This approach assumed that the function would be similar before and after MDA, with only changes to some of the parameters after MDA deployment. For these analyses, the entire dataset with both pre- and post-MDA cases was included, and the optimum models were determined simultaneously for pre- and post-MDA data (see Table 2).

**Table 2:** Comparison of the Combined Scenario B models

Scenario B Model functions		MDA interaction	Both pre- and post-MDA	
			RSE	AIC
1	*y* = *a*(1 − *e*^−(*bx* + *ci*)^)	*δ*	0.1224	**− 83.66**
		*N*	0.1279	− 77.91
2	*y* = *a* − *be*^−(*cx* + *di*)^	*δ*	0.1219	− 83.22
		*N*	0.1242	− 80.83
3	*y* = *ae*^−*b*/(*x* + *ci*)^	*δ*	0.1264	− 80.16
		*N*	0.1353	− 79.91
4	*y* = *ax*^*b* − *ci*^	*δ*	0.1257	− 79.50
		*N*	0.1260	− 70.60

Combined Scenario B models evaluated, with their corresponding RSE and AIC values. The *y* variable represents the predicted antigen estimates, while *x* is the Mf prevalence as the independent variable. *δ* is the presence of MDA while *N* is the rounds of MDA (represented by the independent variable *i*). The number in bold represent the lowest AIC value from all models in this scenario

The parameters for each of these models were estimated using a nonlinear least square method in R software version 3.4.4. The models in the Differentiated Scenario A considered the MDA interaction (either its presence/absence or the number of rounds) as an indicator, where the parameters were first estimated based on the pre-MDA data (*n* = 49), and then, a scaling factor estimated in each model was re-estimated based on the post-MDA data (*n* = 16). In the case of models in the Combined Scenario B, the parameters were estimated from the combined 65 data points but considered the MDA interaction as an independent variable (*i*). We compared the RSE (residual standard error) and the AIC (Akaike information criterion) for all models to evaluate their goodness of fit. The AIC value, in particular, was used to select one optimum model from each scenario. For our standardisation process, we chose only one of these two final models to predict antigen prevalence from Mf-only surveys. This decision was based on which function (1) provided the best AIC, (2) was more consistent across scenarios and (3) was supported by the biggest sample size.

Final standardised prevalence estimates were organised into three prevalence categories, previously suggested by Graves et al. [14]. These antigen cut-off categories are based on a practical understanding of MDA implementation, settings and protocols [14] and included (a) no or very low prevalence below the threshold for initiating MDA (< 1%), (b) low prevalence (between 1 and 5%) and (c) high prevalence (> 5%), in all age groups.

### Spatial analysis

Following a thorough geolocation revision for all surveys included in this study (details described in the Additional file 1: Table S1), we plotted the surveys over a map of Papua New Guinea delineated with district boundaries. The standardised LF antigen prevalence for each survey was displayed and grouped into the three prevalence categories described above. Surveys were also distinguished between three time periods: (a) surveys conducted between 1990 and 1999, (b) surveys conducted between 2000 and 2009 and (c) surveys conducted from 2010 to 2014.

## Results

### Analysis of surveys

**Fig. 1:**
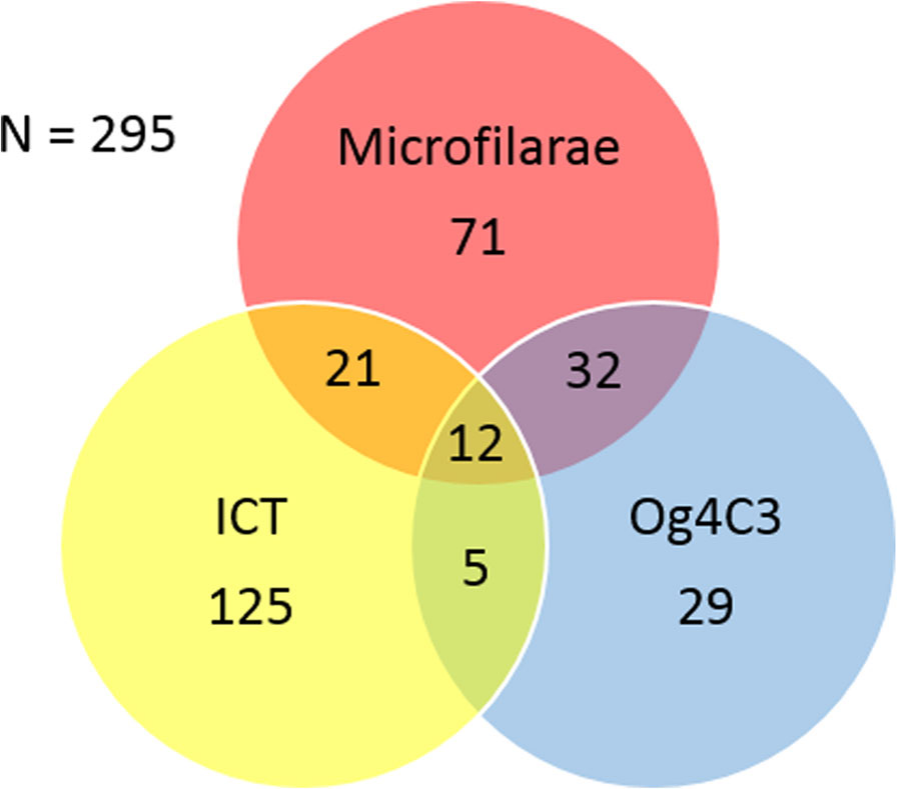
Distribution of diagnostic tests utilised by the surveys included. Values represent the number of surveys that utilised a specific diagnostic or combination of diagnostics

A total of 295 surveys were included in our analysis, of which 117 were conducted between 1990 and 1999, and 125 were performed between 2000 and 2009. The remaining 53 took place between 2010 and 2014. The breakdown of the different diagnostic tests used in these surveys is described in Fig. 1. From the 295 surveys selected, 138 reported Mf results: 71 had Mf results only and 65 reported both Mf and antigen tests together (21 with ICT, 32 with Og4C3 and 12 with both) (Fig. 1).

### Predictive models

We initially considered 65 surveys’ results with both Mf and antigen values to develop our model. ANOVA results showed that there was a significant difference between surveys conducted pre-MDA (*n* = 49) and post-MDA (*n* = 16; median rounds = 1). The median values of Mf prevalence were 0.22 and 0.04 for pre- and post-MDA, respectively (*p* < 0.001). The median values of antigen prevalence for pre- and post-MDA were 0.53 and 0.34, respectively (*p* < 0.01). This interactive effect of MDA on the relationship between tests validated our two modelling scenarios described above. Table 1 shows the results of the four models evaluated under the Differentiated Scenario A.

Table 1 shows how in every instance that the number of MDA rounds was used as an interaction; it resulted in a less fit model than considering the presence/absence of any MDA. This suggests that the occurrence of any MDA plays a greater role in changing the relationship between Mf and antigen prevalence than the number of rounds implemented. From the Differentiated Scenario A, the optimum models were A1 for the pre-MDA data (AIC = − 65.86) and model A4 for the post-MDA condition (AIC = − 14.53).

**Fig. 2:**
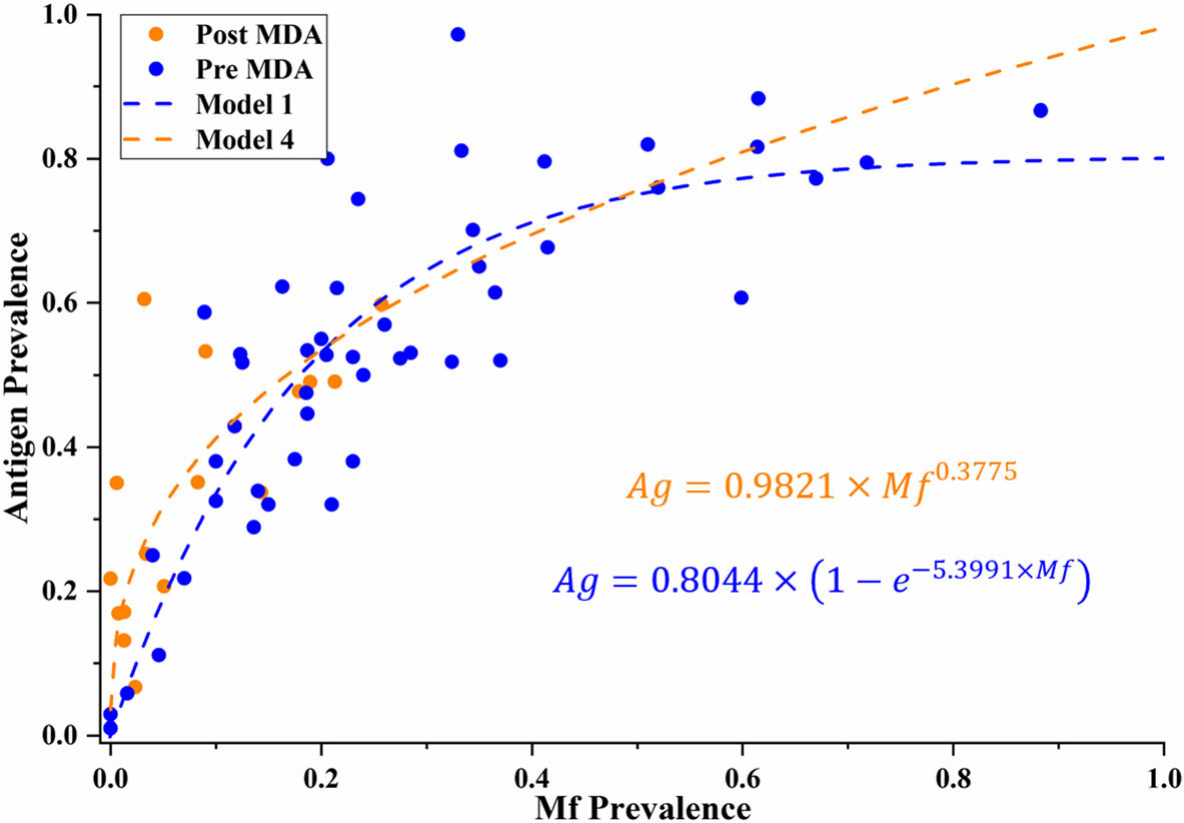
Relationship between antigen and Mf prevalence predicted by models A1 (pre-MDA) and A4 (post-MDA). These combined models result in a “diverging” predictive relationship between diagnostic tests as Mf prevalence increases

Regression analysis of models A1 and A4 produced significant parameters for each function (*p* < 0.0001; please refer to the Additional file 1: Table S2 for details). The resulting curves from models A1 and A4 are described in Fig. 2. This approach resulted in diverging functions, where the pre-MDA curve predicted antigen estimates that reached an asymptote around 0.8 at higher levels of Mf prevalence (Fig. 2), while the post-MDA function reached 1 instead. Confirming predictions at these high Mf values is challenging after post-MDA conditions, due to the effect that MDA has in lowering Mf densities. This was the case with our dataset, where only 16 surveys had post-MDA information for both tests, with the highest Mf prevalence reported as 0.257.

**Fig. 3:**
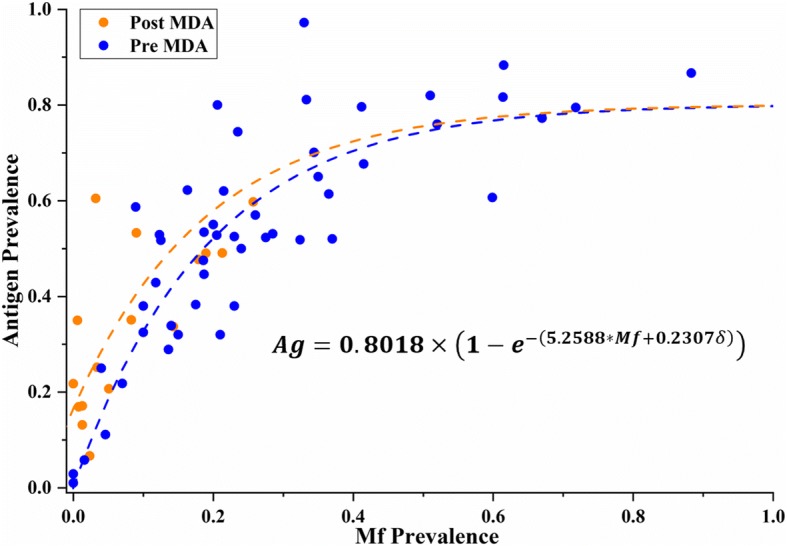
Relationship between antigen and Mf prevalence predicted by model B1. This model suggests a “converging” predictive relationship between diagnostic tests as Mf prevalence increases

Table 2 compares the RSE and AIC values of the four different models considered under the Combined Scenario B. From this group of models, model B1 was the optimum one (AIC = − 83.66). Parameters for model B1 were also significant (*p* < 0.01; please refer to the Additional file 1: Table S3 for details). Figure 3 depicts model B1 with its two corresponding curves. These two functions are a product of the independent variable (*i*) changing from 0 to 1 under pre- and post-MDA conditions, respectively. In this case, the pre-MDA curve is always higher than the post-MDA curve, especially at low Mf levels (Mf < 0.5). This difference decreased as Mf prevalence increased, with both curves converging around a predicted antigen value of 0.8, similar to model A1.

**Fig. 4:**
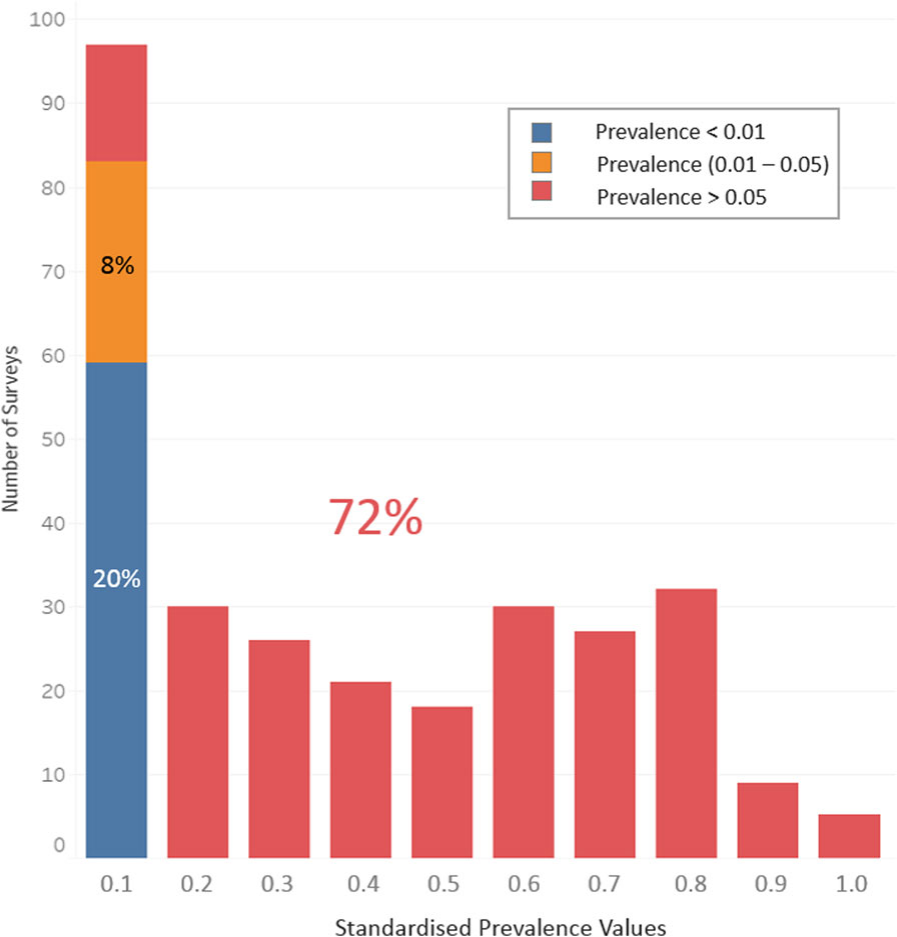
Distribution of surveys according to their standardised prevalence values. Seventy-one out of 295 surveys had their prevalence values predicted using model B1. The three colours shown represent each of our prevalence categories: blue—no prevalence (< 0.01), orange—low prevalence (0.01–0.05) and red—high prevalence (> 0.05), with their respective percentages

We compared the predictions of antigen prevalence from Mf estimates made by models A1 and B1 and found that they were closely aligned with each other. Although model A1 predictions were slightly higher than the model B1 predictions, the difference was within 1% in all cases (*n* = 71 Mf-only surveys). Differences in the predictions between models A4 and B1 were not as closely aligned, however, with up to a maximum of 4.5% difference at Mf levels between 0.2 and 0.3 (these differences are graphically explored in Additional file 1: Figure S1A and B). The similarities in functions and estimated predictions between model A1 (*n* = 49) and model B1 (*n* = 65) lead us to believe that the divergent prediction of model A4 may be a result of the small sample size used (*n* = 16), with no Mf values above 0.3 after MDA. Thus, considering the ample sample size and low AIC value of model B1 (Fig. 3), we decided to use this converging model to predict antigen values for Mf-only surveys (*n* = 71) as part of our prevalence standardisation process. In addition, model B1 produced very reliable predictions at low prevalence levels (< 10%), a critical range for decisions to implement or cease MDA [10]. Final standardised prevalence values for all surveys are summarised in Fig. 4.

Figure 4 shows that a third of the surveys conducted in PNG reported a prevalence under 0.1, while less than 4% of surveys reported prevalence higher than 0.8. A closer look at our three prevalence categories shows that at least 20% of these surveys reported prevalence estimates below 0.01, while roughly three fourths of the surveys described high levels of prevalence, above 0.05 (Fig. 4).

### Risk mapping LF distribution in PNG

**Fig. 5:**
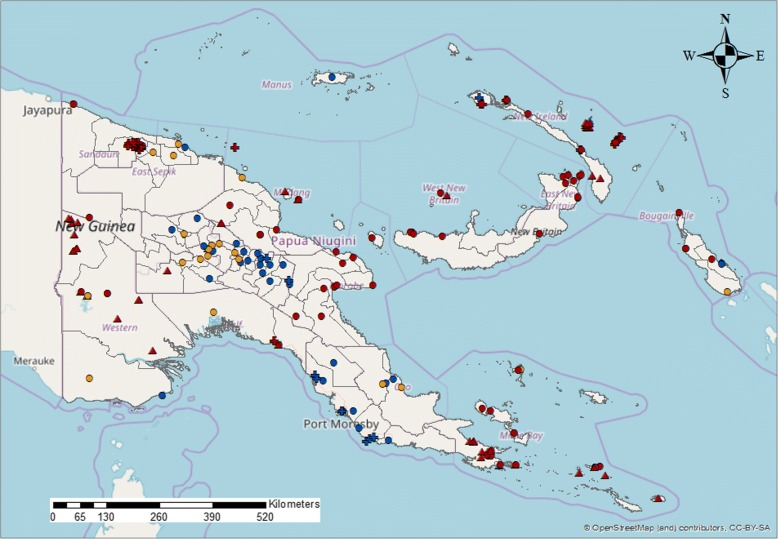
Distribution from surveys conducted: (a) between 1990 and 1999 (triangles), (b) between 2000 and 2009 (circles) and (c) between 2010 and 2014 (crosses). No or very low (< 1%), low (1 to 5%) and high (> 5%) prevalence categories are represented by blue, orange and dark red, respectively

The spatial locations of surveys in PNG over time show variation in the general areas where these studies were conducted during the decades covered in our analysis (Fig. 5). The decade between 2000 and 2009 in particular shows survey efforts spread all throughout the country, with low to no prevalence detected in the central region of PNG. Figure 5 is the most detailed standardised risk map of LF using surveys’ point locations for the entire country to date.

## Discussion

The elimination of lymphatic filariasis in PNG remains a daunting enterprise that requires coordinated international support, as well as national commitment and clear planning. The GPELF has developed a set of guidelines, the initial stage of which requires a comprehensive mapping of LF prevalence in the country. Unfortunately, efforts to develop this spatial undertaking have been patchy and non-encompassing. This has led to inaccurate perceptions of the extent of the disease, as well as misinforming the programme on the true nature of the challenges ahead. Our study aimed to assess this situation and provide an improved and detailed account of the prevalence distribution of this disease in PNG.

Risk mapping usually requires coordinated prevalence studies with the intent of obtaining a homogenous survey effort across the country within a singular timeframe [10]. Prevalence surveys of LF in PNG are heterogeneous in nature, occurring over a span of 30 years, with localised research sites and varied diagnostic methods. Our first challenge was to standardise these different diagnostic results, by producing a predictive model that could provide antigen prevalence values from original Mf estimates. Although a previous study by Cano et al. suggested that this relationship is not predictive [25], our data showed that several predictive models could describe this relationship mathematically and with reasonable precision. We believe that the difference in results between our study and Cano’s team stems from the different approaches used. For instance, while the mentioned paper focused on logistic regression for predicting whether an individual was positive or negative [24], we conducted non-linear analyses on aggregated prevalence estimates from a set of surveys. Other possible differences are discussed further below.

Furthermore, other studies have provided evidence that a relationship between antigen and Mf prevalence exists [26, 29]. Our study, however, is the first one to explicitly describe this relationship using empirical data through two possible types of models. These models differ mainly on their prediction of antigen values after MDA implementation. While models A1 and A4 suggest a diverging effect caused by the MDA interaction at higher levels of prevalence (Fig. 2), model B1 shows a converging result after MDA deployment (Fig. 3).

The shape of the converging curves describing the relationship between antigen and Mf prevalence in model B1 illuminates several aspects of LF biology, transmission and diagnostic performance. Firstly, as suggested in previous studies [26], our model depicts a non-linear relationship between diagnostic tests (Fig. 1). However, model B1 shows a curve that reaches an asymptote at antigen values of approximately 0.8 as Mf prevalence approaches 1. In other words, at high prevalence levels, antigen tests estimate lower prevalence values than Mf tests do. This phenomenon may be explained by the difference in sensitivity between these tests. Previous studies have shown the limitation in sensitivity of antigen tests when detecting LF in patients [23, 30]. While Masson et al. reported an 85.4% sensitivity of Og4C3 tests compared to ICT [30], a study by Gass et al. discussed the different sensitivities of these two antigen tests against Mf results for LF [23]. This latter study reported ICT and Og4C3 sensitivities of 76% and 87%, respectively, against Mf tests [23], suggesting that their sensitivities are not too different. In fact, the resulting averaged antigen sensitivity compared to Mf detection can be calculated as 81.5%, which is highly comparable to predictions from model B1. This may be a result of combining both ICT and Og4C3 in our analyses. Thus, model B1 (and model A1) may be reflecting these sensitivity differences [23], by predicting antigen values of approximately 0.8 when Mf prevalence approaches 1 (Fig. 3).

Another illuminating characteristic of model B1 is that it predicts both pre- and post-MDA curves converging at high prevalence levels (Fig. 3). This could be a product of the effect of MDA on Mf densities in individual patients. The drugs used in an MDA are very effective at dramatically reducing the density of Mf (especially at low density levels) but less effective at permanently killing all adult worms in all individuals (especially in higher density infections) [31]. As infected individuals will have on average lower Mf densities in their blood at low levels of Mf prevalence (and vice versa) [3], we would expect MDA to potentially reduce patients’ Mf density to undetectable levels in such cases. As residual antigen from adult worms would remain circulating in the blood stream after MDA, the ratio between antigen and Mf prevalence would be relatively higher at lower Mf prevalence levels (< 0.3) as shown in model B1 (Fig. 3). However, at higher levels of Mf prevalence, MDA may not completely eliminate Mf densities, rendering Mf easier to detect. As a result, the ratio between antigen and Mf prevalence will decrease as Mf prevalence increases, resulting in similar ratios to pre-MDA surveys. Model B1 (Fig. 3) shows this exact phenomenon, with the pre- and post-MDA curves originally diverging, but converging as Mf increases, and it is thus applicable under both conditions.

**Fig. 6:**
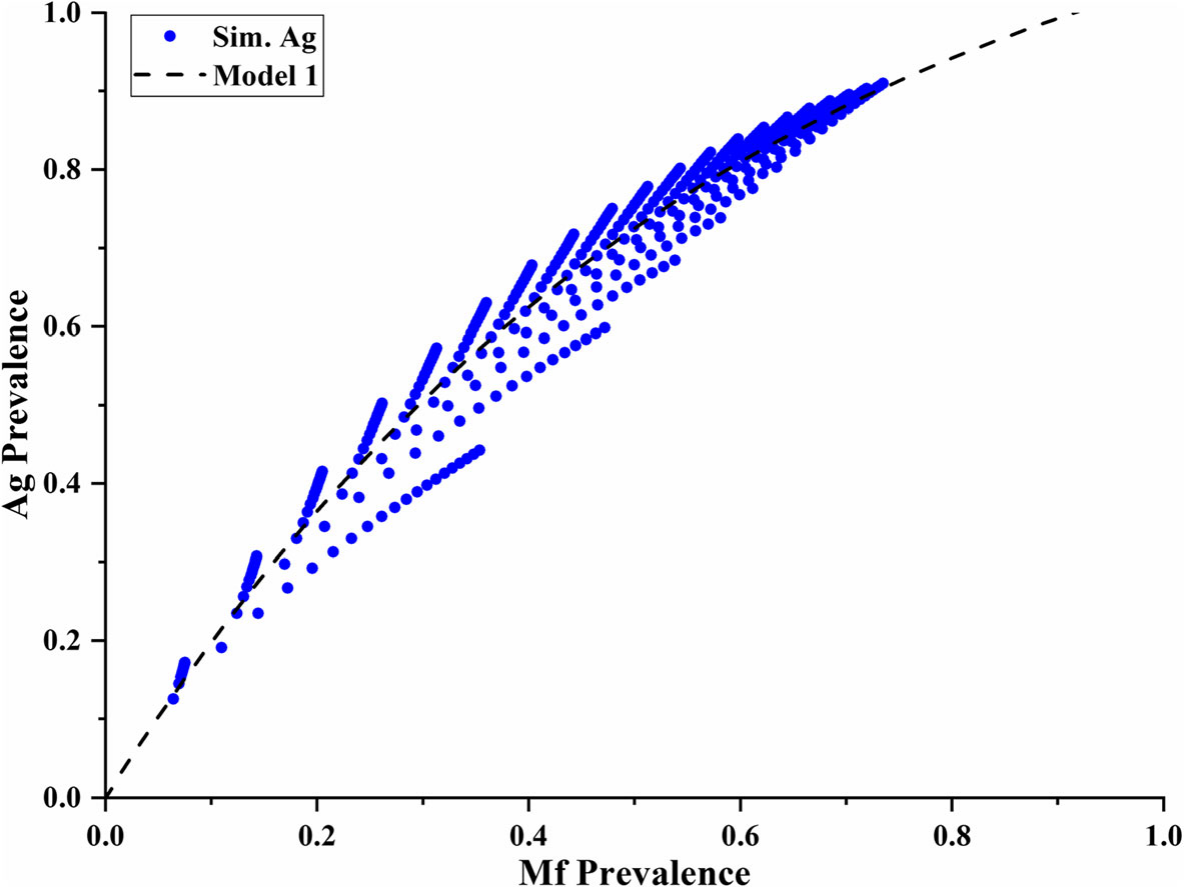
Graph showing the relationship between Mf and antigen prevalence values based on calculations using the model suggested by Irvine et al. [26]. Our parameters’ assumptions were alpha = 0.50 (production rate of mf), m (0.2–4.0), kw (0.2–4.0) and phi (sensitivity, 0.97)

Model B1’s ability to predict the effect of MDA on different Mf densities, as well as reflecting sensitivity differences between tests, supports its use as a predictive model for standardising prevalence values from heterogeneous surveys. However, model A4 deserves a closer look. A previous study by Irvine et al. also tried to describe the relationship between Mf and antigen test results and how it would change after MDA implementation [26]. Initial parameters for their model were based on historical data, while the predicted values for each of the test types were focused on the same area, in an attempt to evaluate the effectiveness of MDA implementation [26]. Irvine and colleagues provided evidence that a correlation should exist to explain the relationship between tests. We used their model to reproduce this correlation, depicted in Fig. 6. Here, we see that the predicted antigen values converge as Mf prevalence approaches either 0 or 1 (Fig. 6).

Figure 6 suggests that, in our interpretation of Irvine et al.’s model, antigen prevalence should always be higher than Mf prevalence (Ag > Mf). This theoretical approach leads to an asymptote only once these two prevalences become equal (at Ag = Mf = 1), which is similar to model A4. Both this theoretical model and model A4 depict the antigen/Mf relationship under post-MDA conditions, suggesting that perhaps under certain circumstances MDA may create a diverging effect as shown in Fig. 2. However, this divergence can also be a result of using a very small sample size of post-MDA surveys in our study. Future research aimed at exploring this possible post-MDA diverging relationship should focus on obtaining values with high Mf prevalence after MDA implementation, which can be highly unlikely.

A number of other sources of error in our study may be responsible for differences between the real relationship between diagnostic tests and our proposed model. Firstly, the method used to detect Mf (amount of blood examined) was not consistent (nor consistently reported) between surveys. Secondly, the sensitivity and specificity of the ICT tests varied over the years, while the Og4C3 ELISA sensitivity and specificity vary according to whether serum or dried blood spots were used [30]. Thirdly, each survey varied in precision due to the range in sample size and the age group of participants tested. Additionally, the information on MDA is available only at a population level, and we do not know whether individuals surveyed after MDA programs had actually participated in the MDA or not. Despite these sources of error between surveys, we were still able to detect a robust relationship between different diagnostic methods to predict antigen values from Mf estimates. This relationship can be used to maximise the utility of serological surveys for spatial risk predictions as well as improving LF transmission models by assisting in predictions of Mf prevalence at different transmission stages, both before and after MDA.

By successfully combining varied survey estimates, we were able to produce a more detailed depiction of LF distribution in PNG, which shows a more complex picture than previously reported. While some previous assessments claimed that PNG had one of the highest LF prevalences in the world [13], one third of the surveys considered in these study reported prevalence results below 0.1 (Fig. 4). In fact, at least a fifth of these surveys described prevalences below 0.01. As such, our study provides a much clearer understanding of the real distribution of LF across PNG.

Among these insights, we can see that the central highland districts of the country show low to no endemicity of the disease—probably due to environmental factors affecting vector distribution, such as altitude and temperature [32]—while high prevalence of LF was located in the lowland and coastal districts. This is particularly the case with the North and South coasts and the Eastern islands. Further survey efforts are needed in underserved areas, such as West Sepik and inland East Sepik Provinces, some provinces in the western highlands, the Southern Highlands Province lowlands and the interior Gulf and Western Provinces, including the Strickland river valley, as well as southern New Britain island.

## Conclusions

Our study confirms the predictive nature of the relationship between antigen and Mf prevalence tests. The models produced included the effect of MDA in this relationship. Model B1 in particular showed a robust goodness of fit and illuminated different aspects related to the sensitivity of the tests and the effect of MDA at different prevalence levels. The predictive nature of model B1 allowed us to aggregate prevalence estimates from assorted LF surveys to more accurately assess the extent of the disease in PNG.

The prevalence distribution of LF across the country is more complex than previously considered. While there are certain provinces and districts showing high levels of prevalence, many other regions of PNG have low to no prevalence that may exclude them from the need to implement MDA. With respect to the GPELF priorities for PNG, our up-to-date risk map based on aggregated surveys identifies high prevalence areas of the country that the programme should prioritise for MDA implementation. Our risk map also identifies underserved survey areas that the programme should further investigate to reach a more detailed understanding of LF distribution in PNG.

Underserved areas, however, may also reflect logistically challenging regions where additional surveys may be unfeasible. Other alternatives to surveillance programs may be required. Further spatial analyses are in progress to develop predictive models based on environmental transmission factors of the disease that can lead to the identification of “hot spots” of potential high prevalence in the country, as well as areas that are very unlikely to be LF endemic, even in the absence of completed prevalence mapping. These spatial tools have been used in previous studies [32, 33] suggesting that their implementation could help other countries in the Asia Pacific region face the logistic challenges of LF risk mapping and eventual elimination.

The elimination of LF remains a global priority, especially in poor communities. Developing new tools and approaches to accurately inform programmes, partners and donors on possible best practices is essential. Our study is a step towards this goal, in the hopes that this information can help in the modelling efforts of LF prevalence around the world and in the identification of potential areas for future localised MDA implementation in PNG.

## Additional file

Additional file 1: **Table S1.** Description of the types of changes made to update survey locations. **Table S2** Estimated parameters—Differentiated Scenario A models. **Table S3** Estimated parameters—Scenario B models. **Figure S1** Comparison of optimum models from Scenario A and B. (a) Plot of the paired predicted antigen prevalence. (b) Difference of predicted antigen prevalence. (DOCX 88 kb)
