# Supplementary material for: Combining different diagnostic studies of lymphatic filariasis for risk mapping in Papua New Guinea: a predictive model from microfilaraemia and antigenaemia prevalence surveys
Source: Trop Med Health. 2018 Dec 4;46:41. doi: 10.1186/s41182-018-0123-8 (PMC6280391; doi:10.1186/s41182-018-0123-8)
Supplement: Supplementary file 1 — Table S1. Description of the types of changes made to update survey locations. Table S2 Estimated parameters—Differentiated Scenario A models. Table S3 Estimated parameters—Scenario B models. Figure S1 Comparison of optimum models from Scenario A and B. (a) Plot of the paired predicted antigen prevalence. (b) Difference of predicted antigen prevalence. (DOCX 88 kb) [file 41182_2018_123_MOESM1_ESM.docx]

**Combining different diagnostic studies of lymphatic filariasis for risk mapping in Papua New Guinea: a predictive model between microfilaraemia and antigenaemia prevalence**

*Alvaro Berg Soto^1^, Zhijing Xu^2^, Peter Wood^3^, Nelly Sanuku^4^, Leanne J. Robinson^4,5^, Christopher King^6^, Daniel Tisch^7^, Melinda Susapu^8^ and Patricia M. Graves^3^*

*Supplemental methods: Geolocation revision*

We reviewed the spatial coordinates for each of the surveys that had previously been geolocated at the district level. However, not all surveys accurately reported their exact geolocation. In some cases, surveys only reported the village, province or district in which they were conducted, and therefore, location reliability ranged from 100 meters to 100 km. In other cases, surveys provided inaccurate coordinates for the stated facility where they were supposedly conducted, requiring revision. Finally, some reported locations wrongly placed surveys at sea, far from the actual PNG coastline. Thus, a thorough geolocation revision was conducted during a period of three months between 2014 and 2015 for all survey records, and improvements were performed when necessary. Spatial information used for this purpose included ESRI satellite data, Mapcarta, Google maps; online and published maps including <https://biostat.wustl.edu/dolf/wp-content/uploads/8.2-DOLF2010PNGsummary.pdf>; and current geographic information compiled and managed by the Papua New Guinea National Agriculture Research Institute (NARI) and University of Papua New Guinea.

Due to the different ways in which the collected surveys reported their field sites, a specific approach was used for each of these instances, with aid from the databases mentioned:

1. In cases where a survey conducted its diagnostics in one single village or a facility (i.e. school) its location was confirmed or changed.
2. In cases where multiple villages were surveyed but only one prevalence value reported, the location was estimated to be at the centre of the total area covered by these villages.
3. In cases where only the district was mentioned and no villages specified, the district capital was chosen as its location, as it was the most likely base of operations for these surveys.
4. When coordinates stated for the corresponding survey facility were inaccurate, satellite imagery and/or NARI data were used to find the facility and assign an updated location.
5. Surveys reported at sea were moved back to the coastline, to a more relevant and accurate location.

As a result, 154 of 295 (52%) survey locations were updated with improved coordinates based on the sources mentioned above. These updated locations were grouped based on the type of change they underwent and described in Table S1. We also report the median spatial errors for these categories, as these errors are not normally distributed.

**Table S1: Description of the types of changes made to update survey locations**

| **Type of changes made to update locations** | **Number of locations** | **Median Spatial Error (m^2^)** |
| --- | --- | --- |
| Relocated to the district’s capital | 49 | 40000 |
| Updated using University of PNG database | 41 | 5000 |
| At sea, moved back to land | 27 | 2000 |
| Updated using NARI database | 15 | 500 |
| Updated using ESRI satellite data | 12 | 5000 |
| Online documents | 7 | 500 |
| Updated using MapCarta | 3 | 10000 |

*Table S1: Number of survey locations updated (n = 154) according to the types of change applied and their median spatial error in m^2^.*

*Supplemental Results: Estimated parameters for both modelling scenarios*

**Table S2. Estimated parameters – Differentiated Scenario A models**

| **ID** | **Model** | **a** | **b** | **c** | **Interaction** | **Scaling** |
| --- | --- | --- | --- | --- | --- | --- |
| 1 | $y=a\left( 1-e^{-bx} \right)$ | 0.8044*** | 5.3991*** | - | $\delta$ | 6.5860*** |
|  |  |  |  |  | $N$ | 5.5950*** |
| 2 | $y=a-be^{-cx}$ | 0.8212*** | 0.7738*** | 4.8275*** | $\delta$ | 5.5570*** |
|  |  |  |  |  | $N$ | 4.6580** |
| 3 | $y=ae^{-b/x}$ | 0.8719*** | 0.0917*** | - | $\delta$ | 0.0636** |
|  |  |  |  |  | $N$ | 0.0458** |
| 4 | $y=ax^{b}$ | 0.9821*** | 0.4072*** | - | $\delta$ | 0.3775*** |
|  |  |  |  |  | $N$ | 0.3255*** |

Significance level ‘***’ 0.001 ‘**’ 0.01 ‘*’ 0.05

The underlined parameters are the scaling factors.

**Table S3. Estimated parameters – Scenario B models**

| **ID** | **Model** | **Interaction** | **a** | **b** | **c** | **d** |
| --- | --- | --- | --- | --- | --- | --- |
| 1 | $y=a\left( 1-e^{-(bx+ci)} \right)$ | $\delta$ | 0.8018*** | 5.2587*** | 0.2307** | - |
|  |  | $N$ | 0.7896*** | 5.6072*** | 0.0651** | - |
| 2 | $y=a-be^{-(cx+di)}$ | $\delta$ | 0.8331*** | 0.7596*** | 4.3867*** | 0.1464. |
|  |  | $N$ | 0.8466*** | 0.7278*** | 4.0238*** | 0.0208 |
| 3 | $y=ae^{-b/{(x+ci)}}$ | $\delta$ | 0.8548*** | 0.0917*** | 0.0513*** | - |
|  |  | $N$ | 0.8206*** | 0.0786*** | 0.0121*** | - |
| 4 | $y=ax^{b-ci}$ | $\delta$ | 0.9686*** | 0.3981*** | 0.0263 | - |
|  |  | $N$ | 0.9625*** | 0.3893*** | 0.0039 | - |

Significance level: ‘***’ 0.001 ‘**’ 0.01 ‘*’ 0.05

*Supplemental Results: Estimated parameters for both modelling scenarios*


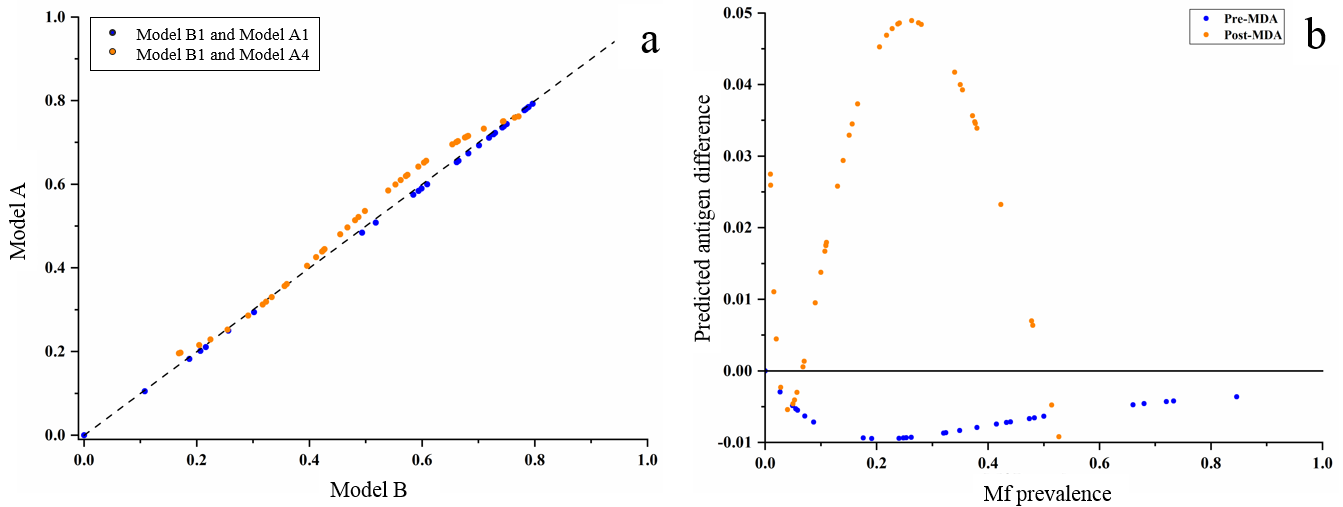


Figure S1: Comparison of optimum models from Scenario A and B. (a) Plot of the paired predicted antigen prevalence. (b) Difference of predicted antigen prevalence.
